# Recent advances and applications of optical coherence tomography angiography in diabetic retinopathy

**DOI:** 10.3389/fendo.2025.1438739

**Published:** 2025-04-16

**Authors:** Qing Zhang, Di Gong, Manman Huang, Zhentao Zhu, Weihua Yang, Gaoen Ma

**Affiliations:** ^1^ Department of Ophthalmology, The Third Affiliated Hospital of Xinxiang Medical University, Xinxiang Medical University, Xinxiang, Henan, China; ^2^ Department of Ophthalmology, The First Affiliated Hospital of Hainan Medical University, Haikou, Hainan, China; ^3^ Shenzhen Eye Hospital, Shenzhen Eye Medical Center, Southern Medical University, Shenzhen, Guangdong, China; ^4^ Zhengzhou University People’s Hospital, Henan Eye Institute, Henan Eye Hospital, Henan Provincial People’s Hospital, Zhengzhou, Henan, China; ^5^ Department of Ophthalmology, Huaian Hospital of Huaian City, Huaian, Jiangsu, China

**Keywords:** optical coherence tomography angiography, diabetic retinopathy, grading, lesion recognition, artificial intelligence

## Abstract

**Introduction:**

Optical coherence tomography angiography (OCTA), a noninvasive imaging technique, is increasingly used in managing ophthalmic diseases like diabetic retinopathy (DR). This review examines OCTA’s imaging principles, its utility in detecting DR lesions, and its diagnostic advantages over fundus fluorescein angiography (FFA).

**Methods:**

We systematically analyzed 75 articles (2015–2024) from the Web of Science Core Collection, focusing on OCTA’s technical principles, clinical applications in DR diagnosis, and its use in diabetes mellitus (DM) without DR and prediabetes. The use of artificial intelligence (AI) in OCTA image analysis for DR severity evaluation was investigated.

**Results:**

OCTA effectively identifies DR lesions and detects early vascular abnormalities in DM and prediabetes, surpassing FFA in noninvasiveness and resolution. AI integration enhances OCTA’s capability to diagnose, evaluate, and predict DR progression.

**Discussion:**

OCTA offers significant clinical value in early DR detection and monitoring. Its synergy with AI holds promise for refining diagnostic precision and expanding predictive applications, positioning OCTA as a transformative tool in future ophthalmic practice.

## 1 Background

Optical coherence tomography angiography (OCTA) is a noninvasive imaging technique that captures detailed images of the retinal and choroidal microvasculature. This method exploits the reflectivity of laser light from the surface of moving red blood cells, Although OCTA cannot directly display the vascular structures, it can detect the presence of blood flow in different regions and layers of the retina (1) Optical coherence tomography (OCT), another retinal imaging technique, uses multiple A-scans to generate a B-scan, providing valuable information on the retina’s structural characteristics in a cross-sectional view (2). OCTA detects and compares changes in blood flow by taking multiple images at different time points, rather than directly measuring blood flow velocity. These time-series images enable OCTA to distinguish regional variations in different blood flow rates (3).

Diabetic retinopathy (DR) is the most common and significant ocular complication among individuals with diabetes. Approximately one-third of diabetics show signs of DR, with some experiencing vision-threatening retinopathy or macular edema (4). DR results from diabetes-related damage to the eye’s tiny blood vessels and can progress from non-proliferative diabetic retinopathy (NPDR) to proliferative diabetic retinopathy (PDR). Diabetic macular edema (DME), characterized by increased blood vessel permeability, thickening, and hard exudates in the macula, frequently occurs regardless of the DR stage (5, 6). OCTA, an emerging imaging modality, enables the detection of effects in various layers of the retina in DR, the impact of different treatment modalities on retinal microvasculature and blood flow, and the correlation between functional levels and anatomical and vascular indicators. As a widely used method, OCTA aids in diagnosing DR and its complications, assisting in DR grading and early detection, particularly in diabetic patients who have not yet developed the condition (7). OCTA’s imaging modality can reveal more profound changes that ophthalmologists’ fundoscopy may not detect, helping predict and detect pre-diabetic retinopathy stage changes. In clinical applications and DR research, OCTA can identify promising and sensitive biomarkers under different modalities, offering new insights into the early-stage pathophysiology and treatment and screening of DR (8).

## 2 History of OCTA

Since its introduction in 2014, OCTA has gained significant traction in clinical practice and has witnessed extensive utilization for various ocular diseases (9). The two most commonly employed OCTA devices are Spectral-domain OCTA (SD-OCTA) and Swept-source OCTA (SS-OCTA), both utilizing Fourier domain detection techniques. However, in SD-OCTA instruments, a broadband near-infrared superluminescent diode serves as the light source, currently operating at a center wavelength of approximately 840 nm, with a spectrometer as the detector. With the advancement of the SD-OCTA device, the clinical application of the prototype 1.0 micrometer SD-OCTA has also been reported. In contrast, SS-OCTA instruments adopt a tunable swept laser with a central wavelength of around 1050 nm and a single photodiode detector (10). SS-OCTA offers the advantage of faster scanning speed compared to SD-OCTA, enabling denser scan patterns and wider scan areas within a given acquisition time. Additionally, the use of longer wavelength in SS-OCTA leads to reduced sensitivity roll-off and improved light penetration through the retinal pigment epithelium (RPE) for better detection of signals from deeper layers. Moreover, the longer wavelength used in SS-OCTA ensures greater safety for the eye, allowing for higher laser power to be employed. The combination of higher power and reduced sensitivity roll-off enhances the ability to detect weaker signals from deeper layers, thus overcoming the barrier posed by the RPE in SS-OCT systems (11). The OCTA algorithm segments the resulting image (ranging from 3 mm^2^–12 mm^2^) into four zones, as per the standard: the superficial retinal plexus, deep retinal plexus, outer retina, and choriocapillaris. When applied to an optical disk, this encompasses the entire depth of the disk (12). While ultra-wide-field OCTA (UWF-OCTA) images have been developed and employed in clinical research, their practical clinical application may be limited due to factors such as longer acquisition time and image quality (13).

## 3 Methods of literature retrieval

This paper aimed to present an overview of OCTA for DR applications. We obtained all the literature from the Web of Science Core Collection (WoSCC), a leading global database of scholarly information founded in 1985. The WoSCC contains both authoritative and influential journals from a wide range of disciplines. We used the WoS core set of journals indexed to the Science Citation Index and combined titles and subjects to search for subject terms to maximize accuracy while maintaining search sensitivity. Our search formula was as follows: TS= ((“Optical coherence tomography angiography” and “diabetic retinopathy”) OR (“OCTA” and “diabetic retinopathy”) OR (“Optical coherence tomography angiography” and “DR”) OR (“OCTA” and “DR”)) OR TI=((“Optical coherence tomography angiography” and “diabetic retinopathy”) OR (“OCTA” and “diabetic retinopathy”) OR (“Optical coherence tomography angiography” and “DR”) OR (“OCTA” and “DR”)) AND (DT==(“ARTICLE”) AND LA==(“ENGLISH”)). Within our search formula, TS=Topic, TI=Title, DT=Document type, LA=Language. We restricted the documents to essays and review papers, limited the language to English, and excluded documents outside the year of the OCTA application.

**Figure 1:**
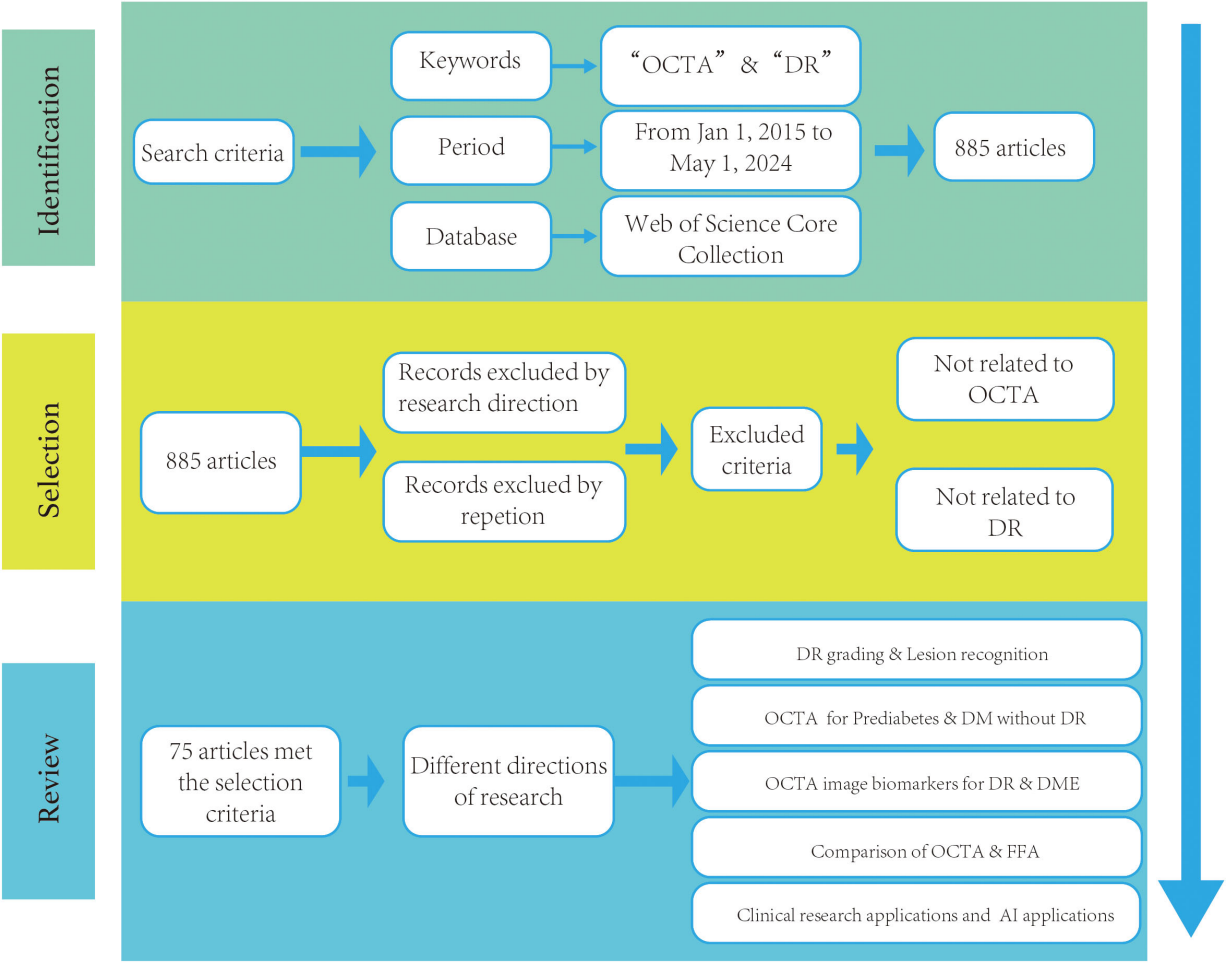
The literature search and review roadmap.

A search of literatures revealed a total of 885 articles published in the field over the last decade. 75 articles were mentioned in this review after being screened by ophthalmic imaging specialists and retinal disease specialists. In this review, many research directions within the field of OCTA in DR applications are mentioned. The Literature Search and Review Roadmap is shown in **Figure 1**.

## 4 Basic imaging features of OCTA

OCTA can produce a signal that reveals the impact of motor blood flow on retinal and choroidal vessels, providing a clear view of the superficial capillary network (SCN) in the ganglion cell layer (GCL), deep capillary network (DCN), and choriocapillaris (CC) in the outer plexiform layer. With its high sensitivity and specificity, OCTA effectively detects neovascularization even in the presence of blood flow (14). OCTA enables the detection of blood vessel shape and distribution in various retina regions, the mean artery count is 7.0 ± 1.2, and the mean vein count is 6.9 ± 1.2 in the 3 mm × 3 mm retina OCTA (15, 16).

OCTA’s capabilities extend beyond the detection of neovascularization, as it also allows for the measurement of the foveal avascular zone (FAZ) size and identification of areas without blood flow in patients who exhibit no visible clinical symptoms. Additionally, OCTA facilitates a more detailed examination of the choroid’s perfusion status, identification of choroidal capillaries, and detection of neovascular complexes in certain non-exudative lesions (17).

## 5 The application of OCTA in DR

### 5.1 DR grading

DR, a potentially vision-threatening condition, poses three primary threats to visual health: vitreous hemorrhages resulting from neovascularization, retinal detachment that can cause substantial vision loss, and localized damage to the macula or fovea, leading to the loss of central vision (4). Historically, the classification and grading of DR severity have been based on visible signs of increasing severity under the color fundus photograph, progressing from no retinopathy to non-proliferative or pre-proliferative stages, and finally to advanced proliferative disease. The grading of DR is crucial in guiding and preventing clinical management of the disease. Precise DR grading can assist patients in better managing the condition, thereby reducing the risk of vision loss. However, this grading system may not accurately represent functionally severe disease, as maculopathy with significant visual loss can occur with moderate ophthalmoscopic signs. This has necessitated the need for disease monitoring. By clearly visualizing key DR pathological features—microaneurysms (MAs), retinal nonperfusion, intraretial microvascular abnormalities (IRMAs), and retinal neovascularizations (RNVs)—OCTA has been endorsed in expert consensus guidelines as a critical adjunct assessment tool for clinical staging and treatment decision-making in DR management (18, 19).

### 5.2 Lesion recognition

**Figure 2:**
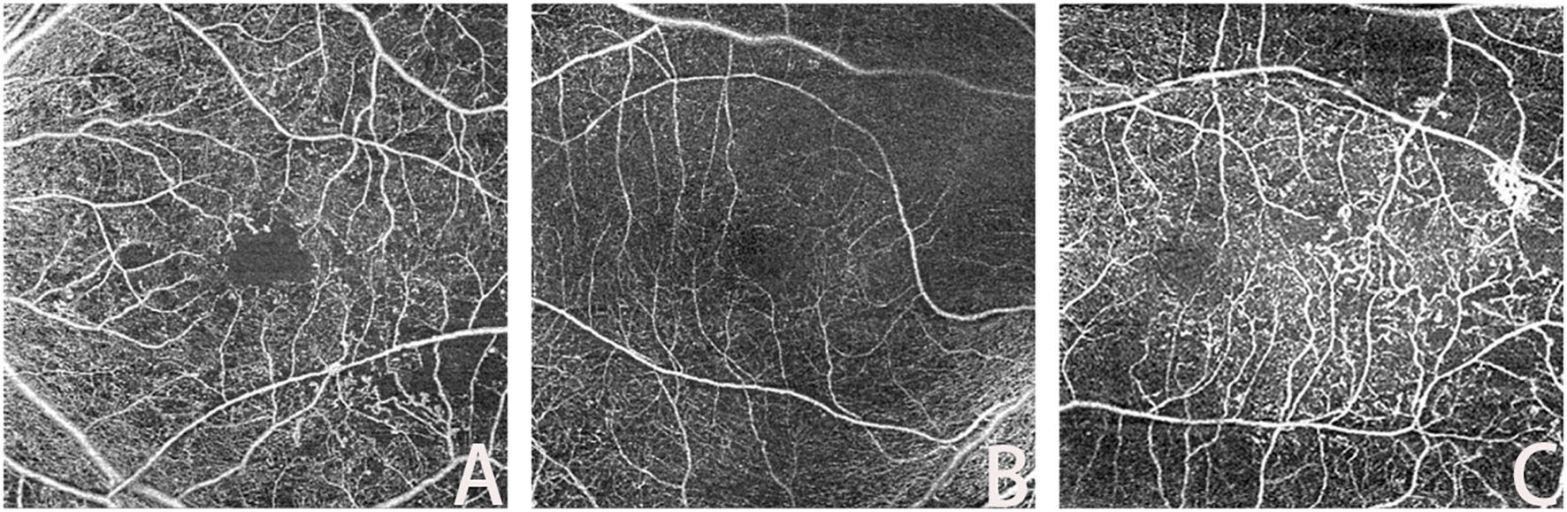
Lesions in OCTA (**(A)**: MA; **(B)**: IRMAs; **(C)**: RNV).

OCTA is employed in clinical practice for the diagnosis of DR, frequently to identify the characteristic lesions of various stages of DR. This complements the diagnosis and grading of DR, such as MAs, IRMAs, and RNVs, as shown in **Figure 2** above.

#### 5.2.1 Microaneurysm

Retinal microaneurysms (MAs) serve as an early symptom of DR, with the type of MA correlating with visual acuity, DR duration, and severity (20). The identification and study of MAs are of interest because their morphological features and turnover are associated with the risk of DR complications and adverse visual outcomes. OCTA techniques can reliably classify retinal MAs (21). Studies have identified various morphological types of MAs, such as focal bulge, saccular/pedunculated, fusiform, and mixed types, and found that these different typologies may correspond to DR progression at varying stages. This understanding allows for the assessment of the relationship between MAs and the progression of clinical stages of DR.

#### 5.2.2 Intraretinal microvascular abnormalities

Intraretinal microvascular abnormalities (IRMAs) were first observed in the eyes of diabetic patients with severe NPDR and PDR. It was initially unclear whether these vascular anomalies indicated the development of new intraretinal blood vessels or the expansion of existing ones (22). IRMAs are characterized by abnormal branching or dilation of existing retinal capillaries, and their presence correlates with the severity and progression of DR (23). These abnormalities may be a consequence of ongoing ischemia.

OCTA images reveal IRMAs as abnormal, branching, and dilated retinal vessels that do not extend into the vitreous cavity (24). OCTA enables the visualization of morphological changes in IRMAs both before and after pan-retinal photocoagulation (PRP), which aids in classifying IRMAs into more specific types. Monitoring these morphological changes in IRMAs can facilitate the early detection of severe signs of DR, potentially leading to earlier treatment interventions (25).

#### 5.2.3 Retinal neovascularization

Retinal neovascularization (RNV) is a critical clinical feature of PDR, representing a pathophysiological alteration resulting from retinal ischemia in DR. This abnormal formation of blood vessels signifies the progression of DR to PDR (26). RNV increases the risk of severe vision loss due to complications such as vitreous hemorrhage or traction retinal detachment. Early detection and timely treatment of RNV are crucial for preventing disease progression and subsequent vision loss (27).

Ophthalmologic fundoscopy can reveal RNV as irregular red blood columns in the optic nerve head and retina. However, the direct visualization of neovascularization is limited by the retinal structure and laser spots formed after treatment. Although OCTA has poor ability to detect NVD or NVE by the lack of detection of leakage from RNV. On OCTA images, the most common neovascular lesions are irregular hyperplasia of fine blood vessels, while some RNVs appear as filamentous neovascular loops (28).

The identification of characteristic lesions of DR, as described, often relies on OCTA as complementary information for DR diagnosis. RNV identification was rigorously performed using B-scan flow analysis, where neovascularization confirmation required detectable flow signals above the internal limiting membrane (ILM), thereby distinguishing true RNV from IRMA. Nevertheless, OCTA has limitations; it cannot detect hard exudates, which are due to the extravasation of lipids and proteins following the breakdown of the blood-retina barrier, nor can it accurately identify vessels with low blood flow velocities or hemodynamically abnormal capillaries.

### 5.3 OCTA imaging biomarkers for diabetes mellitus patients without DR

In patients with diabetes mellitus (DM) without DR, OCTA imaging parameters can detect potential microvascular and neurological changes that may not be observable during fundoscopic examination. A prospective study analyzing OCTA data from patients with type 1 diabetes, in conjunction with clinical data, found that metabolic markers and other patient data influenced OCTA results (29). Several studies have indicated that microvascular changes, such as enlargement and remodeling of the FAZ and capillary nonperfusion zone, may begin before the clinical signs of DR become apparent. OCTA is used to identify diabetic patients at risk for retinopathy and to provide rapid, noninvasive screening for diabetes prior to a systematic diagnosis (30). Retinal microvascular abnormalities, including capillary dropout, dilated capillary loops, tortuous capillary branches, patches of reduced capillary perfusion, irregular FAZ contours, FAZ enlargement, and focal or diffuse choriocapillaris flow impairment, have been observed in some diabetic eyes without retinopathy. These abnormalities may serve as early signs of DR and could potentially act as biomarkers for early detection and monitoring of the disease (31).

OCTA metrics have proven valuable in anticipating the advancement of DR in individuals with type 2 DM through the assessment of thickness measurements, retinal nerve fiber layer, and plexiform layer thickness measurements within the ganglion cell layer. Changes in retinal vascular parameters in participants were identified by OCTA measurements of retinal avascular zones, including area, circumference, circularity, vascular density (VD), and macular perfusion (MP) (29, 32, 33). In individuals diagnosed with type 1 diabetes without any signs of DR, it was observed that the VD of the superficial capillary plexus (SCP), deep capillary plexus (DCP), and choroidal ciliary body was lower compared to that of healthy individuals, indicating an earlier impact on both retinal and choroidal circulation before the manifestation of DR (34). Furthermore, patients with diabetes exhibited a reduced vessel density in the superficial and deep retinas surrounding the central fovea (35). However, various studies have reported conflicting findings regarding changes in the size of the FAZ in the early stages of DR. Some studies suggest a significant increase in FAZ size among patients with diabetes (30), while others found no notable differences in FAZ size between the SCP and DCP regions (36). Given the considerable variability in the FAZ region among healthy individuals, its association with DR requires further investigation and verification (37).

In diabetic patients without DR, there is an association between early changes in the morphology of blood vessels around the optic nerve and the density of blood vessels in the retinal choroid capillaries. These changes are linked to a decrease in the thickness of the nerve fiber layer. Notably, alterations in surface vessel density are more commonly observed in the peripapillary region than in the macular area. It is important to note that patients with diabetes may experience early damage to both neurons and small blood vessels, even before clinical signs of DR are present (38). Furthermore, individuals with diabetes exhibit a reduced vascular response to flash stimuli, and there is a decrease in perfusion density in the deep capillary layer in those without DR (37). OCTA imaging reveals damage in the retinal microvasculature, and it has been observed that patients with type 2 diabetes who have microalbuminuria but do not yet have DR exhibit changes in retinal microcirculation. These alterations could potentially serve as an early monitoring tool for tracking microvascular complications in such patients (39, 40).

RNV can arise from IRMAs. Early detection of IRMAs may serve as a reliable method to predict the progression of PDR. Regular use of OCTA to monitor the occurrence of IRMAs may aid in the timely diagnosis of PDR (41). Identifying alterations in OCT and OCTA parameters in specific diabetic patients at an early stage may serve as an indicator of subsequent overt retinopathy (42). A decrease in macular CC perfusion could potentially act as an initial marker for clinically undetectable diabetic vasculopathy (43). The analysis of intercapillary areas (ICAs) involves measuring the distance between surrounding vessels and each pixel in the intercapillary region. Consequently, employing OCTA in DM patients without existing DR holds promise for early detection and prediction of DR development (44). It is imperative to enhance the quantification of retinal ischemia through various perspectives and integrate OCTA imaging into routine clinical and scientific practices. The adoption of standardized and device-independent image analysis methods becomes essential in this regard (45). OCTA has demonstrated remarkable potential in the management of prevalent ocular complications among DM patients. The use of imaging tests for preventing and controlling DR is particularly crucial for DM patients with existing DR.

### 5.4 Prediabetes OCTA imaging biomarkers recognition

Prediabetes, also known as impaired glucose regulation (IGR), is a pathological condition characterized by blood glucose levels that are higher than normal but have not yet reached the diagnostic criteria for diabetes. According to the World Health Organization (WHO), prediabetes is classified into two types: impaired fasting glucose (IFG) and abnormal glucose tolerance (IGT) (46). The risk of DR may be related to prediabetes. During examination by direct ophthalmoscopy, vascular changes, such as a lower arteriole-to-venule ratio and increased retinal arteriole or venular caliber, were found in the retinas of patients with prediabetes, which may be associated with the pre-diabetic state (47). The current application of the OCTA technique for monitoring blood flow in normoglycemia versus prediabetes shows that the paravascular density in the SCP and DCP layers of the retina was reduced in the prediabetes group compared with that in the normoglycemia group (48).

### 5.5 OCTA imaging biomarkers of DR

In the application of OCTA for DR, several image markers can be utilized to predict the severity of the disease and provide complementary information for DR diagnosis and management. These markers include metrics for the FAZ and vascular and perfusion densities, which have been extensively studied as clinically interpretable features to gauge the severity of DR.

#### 5.5.1 The foveal avascular zone

Foveal avascular zone (FAZ) corresponds to the region of the human retina with the highest concentration of cone photoreceptors and oxygen consumption (49). Researchers have extensively studied the correlation between FAZ changes and various ocular diseases (50). As DR progresses, the fovea, which is responsible for central vision, can be affected. In such cases, abnormalities in the size and shape of the FAZ can contribute to vision loss (51). OCTA assessment of the FAZ area in patients with type 2 diabetes enables early detection of macular changes that precede findings from conventional retinography and SD-OCT examinations (52). One study conducted an assessment of neurological dysfunction in pre-diabetic individuals using multifocal electroretinography (mfERG), analysis of neurodegeneration, and measurement of retinal layer thickness with SD-OCT. Additionally, quantitative parameters such as FAZ area, vessel area density (VAD), vessel length fraction (VLF), vessel diameter index (VDI), and fractal dimension (FD) were measured using OCTA. It should be noted that the association between pre-diabetic neurodegeneration and early microvascular damage is not yet fully understood. However, a glycemic threshold was identified for pre-diabetic patients with observable retinopathy (53, 54).

#### 5.5.2 Vessel density

Vessel density (VD) was defined as the proportion of the vascular area in the image to the total measured area and was used to indicate microvascular perfusion (55, 56). The VD must be corrected for the thickness of the retinal layers during analysis, such as the macular ganglion cell inner plexiform layer (35). Moreover, VD is related to scanning signal strength (57). During clinical applications, poor-quality images should be deleted.

#### 5.5.3 Vascular length density/skeleton density

Vascular length density (VLD)/skeleton density (SD) serves as a metric similar to VD. However, because VD only considers the density per unit area of the vascular area, VLD is deemed to be more sensitive than VD to changes in perfusion at the capillary level (3).

#### 5.5.4 Vessel diameter index

The vessel diameter index (VDI) and the average vessel caliber (AVC) were determined by analyzing binarized and skeletonized images. This metric quantifies the average size of blood vessels in terms of their vascular caliber (54), increased VDI may be associated with higher fasting blood glucose levels (58).

#### 5.5.5 Fractal dimension

Fractal dimension (FD), an indicator of vascular network complexity calculated via methods like box-counting or circular mass-radius in OCTA images, reflects the self-similarity of the retinal capillary network, notably in the superficial (SCP) and deep capillary plexus (DCP), and is automatically computed by artificial intelligence (AI) as a potential biomarker for DR progression (59). Research shows FD typically decreases in DR patients compared to healthy controls and diabetics without DR, especially in the DCP, with Singh et al. (2021) noting a significant reduction using the circular mass-radius method (60)., and Zahid et al. (2016) confirming lower FD in DR versus controls and non-DR diabetics (60), suggesting vascular simplification due to capillary dropout. The FD variation pattern differs across DR stages: in diabetic patients without clinical DR signs, FD may align with healthy controls (60), yet a 2022 study found it already lower, hinting at early changes (61); in NPDR, FD drops below normal and non-DR diabetic levels, particularly in the DCP, reflecting capillary dropout-induced simplification (62); in PDR, FD further declines, with DCP FD lower than in NPDR (60), likely due to intensified capillary loss, though neovascular complexity in PDR may not be fully captured by standard FD measures despite new vessel growth.

#### 5.5.6 Other OCTA imaging biomarkers of DR

OCTA has the capability to identify and objectively detect diseases through alternative biomarkers, such as the VDI, vessel perimeter index (VPI), and vessel skeleton density (VSD) (63). These biomarkers have been further detailed in the context of detecting and categorizing DR during regular care for patients with PDR (64). To quantify the extent of retinal ischemia, the intercapillary area (ICA) is analyzed by measuring the distance between each intercapillary pixel and adjacent vessels (45). The capillary nonperfusion area (NPA) serves as a critical biomarker for assessing DR progression by OCTA, with its distribution varying among patients with different severities of DR (65). Certain OCTA biomarkers and parameters are essential assessment criteria in clinical studies of anti-VEGF drugs, although signal attenuation artifacts can pose challenges to accurate quantification (66, 67). Among these biomarkers, the extrafoveal avascular area (EAA) correlates well with DR severity and demonstrates high sensitivity in differentiating between diabetic and healthy populations (68). In the management of diabetic maculopathy, OCTA can detect changes in reflectance at different stages of progression, which aids in understanding the characteristics of microaneurysms and improves the timing of treatment for diabetic maculopathy lesions, facilitating a more individualized treatment plan (69). OCTA’s potential in managing patients with diabetic macular degeneration has been highlighted by a study showing that some patients with early DR macular degeneration (characterized by microaneurysms, leakage, neovascularization, and internal microvascular abnormalities) exhibit more pronounced changes in the FAZ, despite a relatively normal clinical presentation (52).

### 5.6 OCTA in DME

Utilizing OCTA, the identification and structural assessment of DME now extend beyond merely delineation of internal morphological disruptions. This enhanced investigative modality also affords a nuanced in-depth visual interrogation of the macular microvasculature in DME and enables precise monitoring of dynamic alterations using quantifiable OCTA parameters (70).

#### 5.6.1 Diabetic Macular Ischemia (DMI) and its role in DME progression

The progression of DME is intrinsically linked to the development of DMI. Clinically, DMI presents with enlargement and irregularity in the FAZ, along with the loss of retinal capillaries, predominantly in regions not directly bordering the macula (71). Analysis of outcomes through Fundus fluorescein Angiography (FFA) and OCTA revealed consistent observations, thereby substantiating the gradability of DMI via OCTA (72).

#### 5.6.2 Prognostication and quantitative assessment for DME

Underpinning studies on Diabetic Macular Ischemia have elucidated a predictive capacity within OCTA, particularly through the evaluation of SCP and DCP. This allows for the prognostic discrimination of DME’s advancement. Severe presentations of DME exhibit irregular FAZ morphology and extensive vascular damage within the Deep Vascular Plexus (DVP), compounding vision impairment ancillary to escalated degrees of DME severity, expanded FAZ regions, and accentuated central macular thickness (73). Furthermore, VD has been demonstrated to bear potential in managing DME. Among individuals with DME, there exists a statistically significant decrease in DCP’s VD compared to those without evidence of DME (74).

#### 5.6.3 Role of OCTA in anti-VEGF Therapy for DME

OCTA applications within research about anti-VEGF treatment for DME have been pivotal. By quantifying changes within macular microvasculature through OCTA parameters, enhanced insight and evaluation of the anti-VEGF therapy’s efficacy are facilitated. The integrity of the DCP is closely correlated with the treatment outcomes concerning VEGF inhibitors in DME management. The extent of DCP has been identified as a significant predictor of the response to anti-VEGF therapies (75). A comparative analysis between DME patients and those without DME highlights a distinct deterioration in visual function across multiple quadrants for those with DME. Particularly, after treatment with VEGF inhibitory agents, a superior post-treatment visual prognosis is observed in patients with higher visual deficits in both the SCP and DCP (76).

### 5.7 Impact of age on OCTA parameters in DR assessment

In the assessment of DR using OCTA, age significantly influences the baseline values of parameters such as VD, fractal dimension (FD), and NPA. In healthy populations, VD and FD exhibit a physiological decline with age—for instance, macular VD decreases by approximately 1.5-2.0% per decade (77)—whereas in DR patients, pathological changes superimpose upon age-related degeneration, increasing the complexity of clinical interpretation. In younger patients (<50 years), OCTA parameters are more sensitive to early microvascular changes, with VD reductions reaching 10-12% during the NPDR stage (78), though rapid disease progression can confound results. In older patients (≥60 years), vascular network degeneration may obscure early ischemic signs, and systemic factors such as hypertension often exacerbate parameter heterogeneity—for example, peripapillary vessel tortuosity increases by 15% (79).

## 6 Comparison of OCTA and FFA in DR

OCTA is capable of accurately detecting changes in the retinal vasculature, although it has limitations in identifying specific lesions in clinical practice. Primarily, OCTA provides supplementary information for the management and diagnosis of patients with DR. FFA, an essential diagnostic tool for evaluating the clinical fundus characteristics of DR, can detect primary vasculopathy and vascular anomalies, such as venous bead-like changes and retinal microvascular anomalies, during the development of vasculopathy in DR. In cases of retinal nonperfusion, characterized by dark areas surrounded by large blood vessels, neovascularization is often marked by significant leakage of fluorescent dye into the vitreous cavity on FFA (80). The FFA procedure involves injecting a fluorescent dye into the anterior vein, typically via a short posterior ciliary artery, which then reaches the optic and choroid regions 8 to 12 seconds later. The choroid circulation appears as choroidal flush, a patchy, mottled hyperfluorescence, as the choroid lobules fill (81). Retinal circulation occurs 1 to 3 seconds later (11-18 seconds after injection). Early arteriovenous malformation (AVM) is associated with the filling of the retinal arteries, arterioles, and capillaries, followed by an advanced arteriovenous or laminar venous phase as the dye fills the veins in a laminar fashion. After about 10 minutes, the complete emptying of the dye occurs, and during this phase, the optic disc, Bruch’s membrane, choroid, and sclera are stained (82). The normal stages of FFA include: 1. choroidal phase; 2. arterial phase; 3. arteriovenous phase; 4. venous phase; and 5. recirculation phase. The features of DR, as identified by OCTA and FFA, are summarized in **Table 1**.

**Table 1:** Comparison of OCTA and FFA.

Lesion type	Etiology	OCTA	FFA
MA	Elevation of capillary pressure due to loss of perivascular and endothelial cells and smooth muscle cells, etc., leading to focal dilatation of capillaries.	Demarcated, saccular or fusiform shapes of focally dilated capillary vessels in the superficial and deep plexuses.	Early stage shows hyperfluorescent pinpoint dots; late stage shows focal leakage.
IRMA	Remodeling of retinal capillaries due to retinal hypoxia.	Clusters of irregular tortuous vessels or dilation of existing capillaries, without proliferative changes. Intraretinal flow below ILM.	Can be distinguished from neovascularization by no or less profuse leakage on FFA. These vessels do not extend over vascular branches.
RNV	Proliferation of fibroblasts due to hypoxia after localized closure of retinal capillaries.	Lesions show retinal new vessels breaching the ILM.	Early stage is strongly fluorescent abnormal vascular network; late fluorescent leakage is clumped strong fluorescence.
DME	Accumulation of fluid, lipids, and proteins in the retina that occurs after rupture of the blood-retinal barrier.	/	Hyperfluorescent areas.

MA, Microaneurysm; IRMA, Intraretinal Microvascular Abnormalities; RNA, Retinal Neovascularization; DME, Diabetic Macular Edema; FFA, Fundus Fluorescein Angiography.

**Figure 3:**
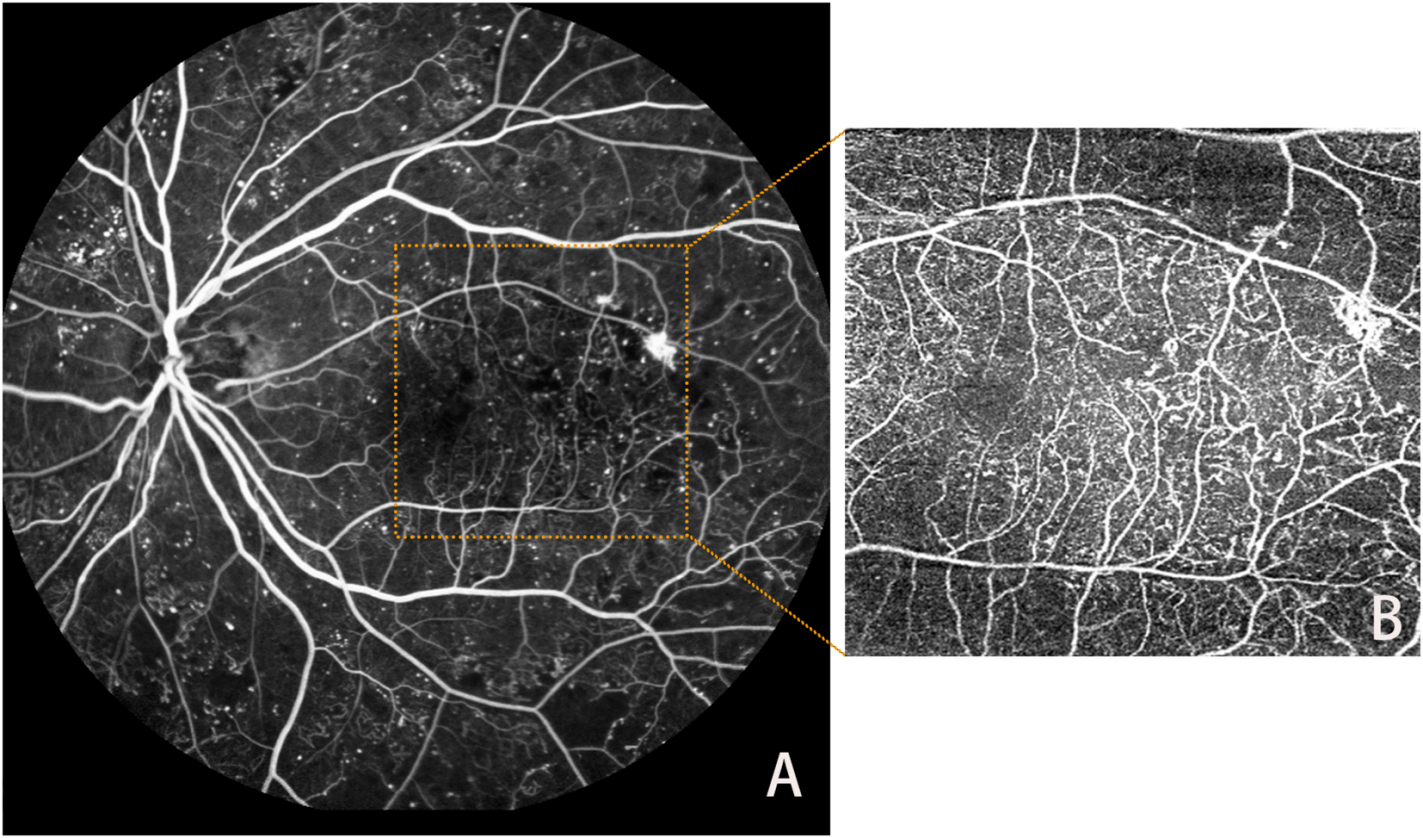
Lesions in FFA **(A)** and OCTA **(B)**.

FFA offers a wider field of view and can dynamically display retinal blood flow. In contrast, OCTA provides a clearer visualization of dynamic blood flow at various retinal levels under different parameters, although it may be slightly less effective in detecting specific lesions with inaccessible or localized blood turbulence (80). The primary clinical disadvantage of FFA is its invasiveness and time-consuming nature. The contrast agent used in FFA can cause several potential side effects, including nausea, vomiting, hives, seizures, allergic reactions, and even death (83). Therefore, the benefits of FFA must be weighed against the potential risks and the availability of less invasive imaging procedures in clinical practice, as presented in **Figure 3** below.

As a noninvasive, dye-free method for ocular vascular visualization, OCTA complements other diagnostic investigations in diagnosing, screening, and managing patients with DR. The advent and development of UWF-OCTA have partially addressed the limitations of OCTA’s small-image recognition area. UWF-OCTA can detect a broader range of retinal images during the actual acquisition process, but its longer image acquisition time may result in more artifacts and lower resolution. Thus, it is crucial to find the optimal balance between scanning area and parameter settings for UWF-OCTA under different circumstances (13, 84).

When assessing the severity of DR, UWF-OCTA has demonstrated promising results in evaluating DR retinal blood flow status. It captures more detailed information about retinal blood flow operations at once compared to standard OCTA due to its larger image range (40, 85). Furthermore, when used as input data for artificial intelligence (AI) analysis, UWF-OCTA reflects the significant potential of integrating AI with clinical applications, as it contains richer image information (86).

## 7 OCTA in DR-related clinical researches

In clinical studies, OCTA imaging modalities facilitate the identification of complications that may lead to retinal ischemia and assess the natural course and progression of retinal ischemia in DR, emphasizing the importance of prevention (87). However, in certain cases where patients have background retinal structures and laser scars, the detailed manifestation of the disease may not be visualized solely through color fundus photograph examination. OCTA has proven effective in accurately detecting various types of neovascularization, particularly beneficial for monitoring patients with a history of retinal surgery (28). OCTA is extensively used in diverse clinical studies, such as assessing retinal inner layer (DRIL) and ellipsoid zone (EZ) losses, leading to the identification of biomarkers that contribute to visual function evaluation in patients’ eyes (88).

The assessment of GCL and inner plexiform layer (IPL) thickness for detecting retinal neurodegenerative changes has revealed a correlation between retinal vascular closure and the advancement of DR severity in patients with NPDR. This suggests that OCTA vascular closure measurements can serve as an indicator of DR progression severity. Post-analysis of OCTA images has shown that retinal neurodegeneration exhibits consistent and stable progression, potentially due to a reduction in vessel density, including vessel closure, caused by alterations in the DCP (89, 90). The analysis of biomarkers, such as VAD, VLF, and VDI, provides quantitative insights into neuronal damage observed in DR patients, indicating that it cannot be attributed solely to microvascular damage (42). The increasing use of OCTA in clinical research underscores its growing significance in advanced ophthalmic studies, including investigating potential retinal microvascular damage caused by different treatment modalities. This technique effectively assesses the number and circulatory condition of peripapillary microvessels, evaluating surgical outcomes and prognoses in clinical settings. For example, OCTA can be used when evaluating the efficacy and follow-up of surgical procedures like pars plana vitrectomy (PPV) combined with ILM peeling for epiretinal membrane (ERM) cases (91). It can also be applied in combination with certain drugs in proliferative DR and various neovascularization-related retinal diseases (92), assessing the efficacy of drug application in DR patients, and comparing the efficacy and safety after different drug applications (93). When applied to DR, OCTA parameters have been found to be good predictors of the efficacy of anti-VEGF drugs (ranibizumab (94), bevacizumab (95)) and also play a role in observing the therapeutic efficacy of other fundus implants (e.g., drug nanoparticles) (96). OCTA has played a crucial role in assessing retinal nerve damage and microvascular changes in several experimental studies (97). Compared to other imaging techniques, such as FFA, OCTA is more accurate in detecting specific lesions in clinical research and has gained broader application due to its noninvasive, user-friendly, and harmless nature.

## 8 AI for OCTA in DR

The diagnosis of DR and the selection of treatment approaches and timing heavily depend on digital imaging data, encompassing OCT and FFA. Targeted feature extraction and objective feature quantification present significant opportunities for biomarker discovery, disease burden assessment, and prediction of treatment response. With the recent advancements in AI in ophthalmic applications, AI-assisted image analysis and OCTA data acquisition, generation, and collection can be technologically integrated. Machine learning-based techniques are particularly well-suited for leveraging OCTA to gather a vast amount of information. AI can transform the generation of OCTA images from a motion-contrast measurement to an image transformation problem, potentially reducing artifacts that are challenging to manage in traditional OCTA image formation (98). For diabetic patients without overt DR signs, OCTA’s quantitative vascular metrics (e.g., vessel density and morphological parameters) serve as tools for early screening and follow-up (99). The application of AI in analyzing and processing various biomarkers derived from OCTA has revealed the potential of specific biomarkers to serve as new imaging biomarkers for the early detection of DR (**Table 2**).

**Table 2:** Performance summary of AI techniques in OCTA application.

Reference	AI Technique Used	Performance Metrics	Journal/Conference
Le D, et al. (2020) (100)	Transfer learning with VGG16 CNN	Cross-val Acc 87.27%, Ext-val Acc 70.83%	Translational Vision Science & Technology
Eladawi N, et al. (2018) (101)	ANN with blood vessel reconstruction	Acc 94.3%, Spec 87%, Sens 97.9%	Medical Physics
Xu X, et al. (2023) (102)	AV-casNet neural network for segmentation	Arteriole Acc 94.2%, Venule Acc 93.5%	IEEE Trans Med Imaging
Afarid M, et al. (2022) (61)	Feature analysis using radiomics features	AUROC 0.87 for FAZ area	BMC Ophthalmol
Schottenhamml J, et al. (2021) (103)	Computational method for segmentation (graph-based)	Mean absolute error: ~0.91 pixel	Biomed Opt Express
Ryu G, et al. (2021) (104).	Deep Learning (CNN)	AUC 0.976, Sens 96%, Spec 98%	Scientific Reports

Acc, Accuracy; Spec, Specificity; Sens, Sensitivity; AUROC, Area Under the Receiver Operating Characteristic Curve; Cross-val, Cross-validation; Ext-val, External validation.

### 8.1 AI for OCTA quantitative parameter analysis

The FAZ, a central avascular region in the macula, typically enlarges in DR due to capillary dropout, with studies showing significantly higher median FAZ area in DR patients, especially in severe NPDR and PDR stages (105). AI, via deep learning (DL) automates FAZ quantification; Guo et al. (2021) demonstrated strong consistency with manual measurements (68), underscoring AI’s precision in measuring FAZ area as a potential DR severity biomarker.

Vessel density, reflecting overall retinal vascular density, decreases in DR progression, notably in superficial and deep capillary plexuses, indicating vascular loss. Research highlights a marked VD reduction with increasing DR severity, particularly post-VEGF inhibitor treatment (106). AI employs DL for vessel segmentation and density quantification, with Wang et al. (2020) validating high consistency with manual measurements (107), affirming AI’s efficacy in assessing VD as a DR progression marker.

FD measures vascular complexity, with varying findings in DR. Some studies report reduced FD in DR patients, reflecting network simplification; Hashmi et al. (2021) found significantly lower FD using the circular mass-radius method (108), supported by Zahid et al. (2019) in the deep capillary layer (60). Conversely, early DR may show FD increases due to abnormal vessels or microaneurysms, though OCTA-specific evidence is limited. AI rarely measures FD directly, relying on image processing algorithms, but may implicitly learn FD-related features for DR classification. These parameters, analyzed via AI, serve as potential biomarkers for DR severity and progression, though FD variability warrants further investigation.

AI enhances DR detection accuracy through parameter analysis. Kim et al. (2021) achieved an AUC of 0.976, sensitivity of 96%, and specificity of 98% using a CNN model (104). Le et al. (2020) reported a cross-validation accuracy of 87.27% with VGG16 transfer learning, dropping to 70.83% in external validation, highlighting generalization issues (100). Eladawi et al. (2018) used support vector machines (SVM) to extract FAZ area and VD features, achieving 94.3% accuracy (101).

### 8.2 Intelligent classification of DR

The rapid advancement of AI has revolutionized DR classification through convolutional neural networks (CNNs) and transfer learning models. Studies demonstrate high diagnostic accuracy, such as Kim et al.’s CNN model achieving an AUC of 0.976 with 96% sensitivity and 98% specificity using 6×6 mm² OCTA images (104). However, generalization challenges persist, exemplified by Le et al.’s VGG16 transfer learning model showing an 87.27% cross-validation accuracy that dropped to 70.83% in external validation (109).

AI algorithms predict DR severity by analyzing biomarkers including NPA, macular ganglion cell/inner plexiform layer thickness, retinal arteriole/venule conditions, and extrafoveal vessel density (102). CNN-based approaches further classify DR into five stages (Normal to PDR) by processing OCTA parameters, enhancing grading precision (85). Despite these advancements, variability in imaging protocols and instrument settings underscores the need for standardized acquisition parameters to ensure broader clinical applicability.

### 8.3 Other AI applications for OCTA in DR

In the application of AI to OCTA images of DR patients, motion correction techniques can be employed to eliminate artifacts caused by eye movements, thereby enhancing the clarity of images, DR characteristic retinopathy, and surrounding features (110). During the collection and processing of information by AI, it is advisable to construct a standard computer-aided diagnostic (CAD) system as defined in each learning model. OCTA, as part of ophthalmic imaging examinations, has demonstrated significant effectiveness in retinal assessment when combined with DL and machine learning (ML) methods, along with other patient imaging data (111). Some studies suggest that collaborative DL can yield comparable outcomes to conventional DL, achieved by utilizing jointly learned models and merging databases for microvessel segmentation and DR classification. These findings highlight the potential applicability of jointly learned models across various domains in OCT and OCTA data (112).

Enhancing the objectivity of OCTA image interpretation in future research, longitudinal tracking, and the integration of computational models to create automated diagnostics and clinical decision-support systems can augment their practical applications. Advances in computational technologies, including DL and radiomics, offer the possibility of developing distinct imaging phenotypes. The construction of these ocular imaging phenotypes can facilitate personalized disease management and increase opportunities for precision medicine. These quantifiable biomarkers and automated methods can be applied to individualized medicine, where treatments are tailored to patient-specific, longitudinally trackable biomarkers, and response monitoring can be achieved with high accuracy. Despite the integration of AI with OCTA image information in DR research, a standardized CAD system remains elusive. The evaluation criteria for learning models across different AI clinical studies are not well-defined. It is crucial to assess these models based on uniform and standardized criteria to enhance the overall proficiency of AI clinical studies in DR applications using OCTA (113).

## 9 Shortcomings of OCTA in DR

OCTA has several limitations, including a limited field of view and an inability to visualize certain DR-characteristic lesions such as vascular leakage, as well as increased susceptibility to artifacts during the technique’s application, such as blinking, motion, and vascular reimaging. Additionally, OCTA cannot detect blood flow below the slowest detectable flow rate (66). Traditional markers for DR diagnostic grading, such as blood-retinal barrier disruption and vascular leakage, lack clear diagnostic markers in OCTA. The standardization of output from different OCTA instruments also significantly impacts the subsequent processing of image data. Current OCTA imaging also faces challenges, such as the inability to detect some ischemic vessels, specific blood flow rate requirements, low imaging efficiency for larger resolution images, and the inevitable formation of noise and artifacts due to subject eye movements during the imaging process. OCTA has a limitation in effectively identifying detailed lesion structures and comprehending the overall architecture of the retinal vasculature in DR.

Further research is needed to determine the utility of OCTA in clinical settings, given its relatively short history of clinical application, and to explore its potential for detailed visualization of the retinal vasculature. Subsequently, OCTA parameters and settings need to be standardized, and standardized guidelines based on the application of OCTA for DR diagnostic grading should be developed. Despite these disadvantages, OCTA offers the advantage of being noninvasive and capable of obtaining volume scans segmented to a specific depth within seconds. Future advancements should focus on obtaining a larger field of view, faster scanning speeds, and higher resolution.

While AI demonstrates high accuracy (100), automation, and potential for early screening in resource-limited settings, its clinical application faces challenges: 1) High-cost dependency on large annotated datasets for training robust models; 2) Limited generalizability, exemplified by Le et al.’s (2020) external validation accuracy of 70.83% (114); 3) Reduced clinical trust due to the black-box nature of DL; 4) Misclassification risks from artifacts (e.g., projection artifacts, motion artifacts); 5) Widespread neglect of age-stratified corrections in current models, leading to misdiagnosis of age-related VD/FD decline as DR progression or underestimation of early pathological signs in younger patients.

Future advancements should prioritize: developing interpretable AI frameworks with age-adaptive feature decoupling to distinguish physiological aging from pathological changes; establishing multicenter age-stratified cohort validation systems and age-specific reference standards; optimizing artifact correction through interdisciplinary collaboration; and promoting standardized CAD systems (115) for unified evaluation. Despite challenges in data scalability, model generalizability, and image quality dependency, the synergy of AI and OCTA holds transformative potential for precise DR management (116). Crucially, AI should be positioned as a clinical aid to enhance diagnostic objectivity, not as a replacement for expert judgment.

## 10 Conclusion

Since the advent of OCTA imaging technology, numerous clinical studies have explored its applications. OCTA is a common noninvasive method for examining retinal diseases, offering simplicity and convenience. Its advantages—noninvasiveness, dye-free imaging, rapid processing, and accurate blood flow localization—have led to its swift adoption in ocular examinations.

Initially, OCTA was primarily an auxiliary imaging tool for DR due to its unique imaging capabilities. However, as clinical studies progressed and the technology evolved, OCTA has increasingly been used to assess retinal nerve and vascular function in DR. It is particularly sensitive in detecting subtle lesions indicative of DR and DM before they manifest. OCTA aids in diagnosing and classifying established DR and can predict and manage retinal conditions in DM patients without DR. It can also identify retinal microcirculation states in prediabetic patients, which is beneficial for community screening and managing DM-related ocular complications.

Recent advances in OCTA for DR have focused on enhancing image resolution and improving software algorithms to better visualize microvascular changes. These improvements allow for earlier DR detection, detailed retinal vasculature mapping, and non-invasive DR progression monitoring. Enhanced OCTA technology also facilitates more accurate treatment efficacy assessments, improving patient management and outcomes. OCTA has the potential to serve as an independent predictor of DR. In clinical applications, OCTA is commonly used as a supplementary diagnostic tool for DR grading. Current technological developments aim to collect high-resolution, wide-field OCTA images faster, leveraging AI’s powerful processing capabilities. Further exploration is needed to standardize OCTA outputs and terminology across different instruments to fully realize its potential in diagnosing, evaluating, and predicting DR.

Although OCTA is not yet a standalone diagnostic criterion for DM in ocular imaging, the emergence of UWF-OCTA and AI integration in ophthalmology has identified more diagnostic and clinically relevant OCTA biomarkers. Despite the lack of standardized grading and interpretation methods for DR assessment, ongoing clinical research and advanced AI models are expected to establish Diagnostic Classification Guidelines for OCTA in DR soon. OCTA will continue to explore new biomarkers and practical applications, playing an increasingly significant role in the prediction, diagnosis, and management of DR across various scenarios.

## Data Availability

The original contributions presented in the study are included in the article/supplementary material. Further inquiries can be directed to the corresponding authors.
